# TCM2COVID: A resource of anti‐COVID‐19 traditional Chinese medicine with effects and mechanisms

**DOI:** 10.1002/imt2.42

**Published:** 2022-08-05

**Authors:** Liping Ren, Yi Xu, Lin Ning, Xianrun Pan, Yuchen Li, Qi Zhao, Bo Pang, Jian Huang, Kejun Deng, Yang Zhang

**Affiliations:** ^1^ Innovative Institute of Chinese Medicine and Pharmacy, Academy for Interdiscipline Chengdu University of Traditional Chinese Medicine Chengdu China; ^2^ School of Healthcare Technology Chengdu Neusoft University Chengdu China; ^3^ School of Life Science and Technology University of Electronic Science and Technology of China (UESTC) Chengdu China; ^4^ College of Medical Technology Chengdu University of Traditional Chinese Medicine Chengdu China; ^5^ College of Food and Biological Engineering Chengdu University Chengdu China; ^6^ Beijing CapitalBio Technology Co., Ltd. Beijing China

**Keywords:** COVID‐19, database, herb, natural product, traditional Chinese medicine

## Abstract

In China, traditional Chinese medicine (TCM) has been widely used for coronavirus infectious disease 2019 (COVID‐19) prevention, treatment, and recovery and has played a part in the battle against the disease. A variety of TCM treatments have been recommended for different stages of COVID‐19. But, to the best of our knowledge, a comprehensive database for storing and organizing anti‐COVID TCM treatments is still lacking. Herein, we developed TCM2COVID, a manually curated resource of anti‐COVID TCM formulas, natural products (NPs), and herbs. The current version of TCM2COVID (1) documents over 280 TCM formulas (including over 300 herbs) with detailed clinical evidence and therapeutic mechanism information; (2) records over 80 NPs with detailed potential therapeutic mechanisms; and (3) launches a useful web server for querying, analyzing and visualizing documented formulas similar to those supplied by the user (formula similarity analysis). In summary, TCM2COVD provides a user‐friendly and practical platform for documenting, querying, and browsing anti‐COVID TCM treatments, and will help in the development and elucidation of the mechanisms of action of new anti‐COVID TCM therapies to support the fight against the COVID‐19 epidemic. TCM2COVID is freely available at http://zhangy-lab.cn/tcm2covid/.

## Highlights

- TCM2COVID documents over 280 TCM formulas (including over 300 herbs) with detailed clinical evidence and therapeutic mechanism information.
- TCM2COVID records over 80 NPs with detailed potential therapeutic mechanisms.
- TCM2COVID launches a useful web server for querying, analyzing, and visualizing documented formulas similar to those supplied by the user (formula similarity analysis).

## Graphical abstract

TCM2COVID documents over 280 traditional Chinese medicine formulas (including over 300 herbs) with detailed clinical evidence and therapeutic mechanism information; TCM2COVID records over 80 natural products with detailed potential therapeutic mechanisms; TCM2COVID launches a useful web server for querying, analyzing, and visualizing documented formulas similar to those supplied by the user (formula similarity analysis).

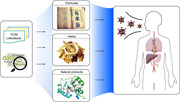

## INTRODUCTION

The coronavirus infectious disease 2019 (COVID‐19) pandemic has lasted for more than 2 years and is a serious threat to global public health [1, 2, 3]. The highly contagious BA.2 sub‐variant of omicron has been surging in many countries, and as of 1 May 2022, over 500 million confirmed cases and over six million deaths have been reported globally [4, 5]. Nevertheless, medical workers and scientists all over the world are working tirelessly to combat the epidemic [5, 6]. In China, traditional Chinese medicine (TCM) has been widely used for COVID‐19 prevention, treatment, and recovery and has played a part in the battle against the disease [1, 7, 8, 9]. A variety of TCM formulas such as “3 medicines (Jinhua Qinggan granule, Lianhua Qingwen capsule/granule, and Xuebijing injection)” and “3 formulations (Qingfei Paidu, Huashi Baidu, and Xuanfei Baidu granule)” have been recommended for different stages of COVID‐19, covering the asymptomatic infection period, clinical treatment period, and recovery period [10, 11]. In addition, some Asian countries such as South Korea and Japan have fully or partially incorporated TCM treatments [12, 13].

**Figure 1:**
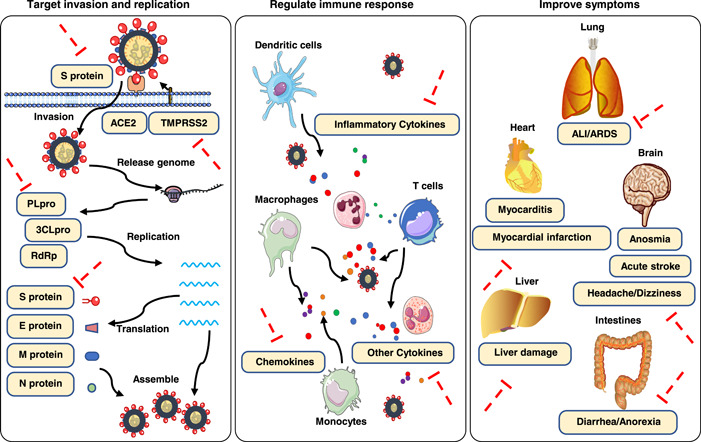
Therapeutic mechanisms of TCM treatments. (1) inhibiting virus invasion and replication by targeting key molecules; (2) regulating immune and inflammatory responses by targeting various immune factors; (3) improving complications and symptoms. TCM, traditional Chinese medicine.

At present, a series of clinical trial studies from multiple hospitals in China have been launched to investigate the efficacy and safety of TCM therapies for COVID‐19. Accumulated clinical evidence shows that TCM has a significant positive impact on COVID‐19 patients, by reducing clinical symptoms, promoting recovery, improving chest images, and inhibiting disease progression [14, 15]. Meanwhile, an increasing number of studies on therapeutic mechanisms have shown that TCM treatment not only targets the virus but also improves the whole body [16, 17, 18, 19]. In addition, many natural products (NPs) and derivatives (such as Proscillaridin A, Scutellarein, Phillyrin, Quercetagetin, and Dihydromyricetin) from TCM herbs have been shown to exhibit activity against SARS‐Cov‐2 by in vitro assays and/or in silico methods (network pharmacology analysis, molecular docking, and virtual screening, etc.) [20, 21, 22, 23, 24, 25]. Summarizing the evidence from current studies, the effects and mechanisms of the TCM therapies mainly include the following: inhibiting virus invasion and replication by targeting key molecules (the S protein, ACE2, 3CLpro, PLpro and RdRp, TMPRSS2, etc.); regulating immune and inflammatory responses by targeting various immune factors; and improving complications and symptoms (Figure 1) [1, 26, 27, 28, 29].

Recently, some studies began to collect the applied formulas of TCM, systematically explore the potential mechanisms underlying the TCM recipes, and screen the key herbs and ingredients for combating COVID‐19 [16, 30]. However, to the best of our knowledge, a comprehensive database for storing and organizing anti‐COVID TCM treatments is still lacking. Herein, we developed TCM2COVID, a manually curated resource of anti‐COVID TCM formulas, NPs, and herbs, which aims to provide a platform for the efficient manipulation, browsing, and analysis of anti‐COVID TCM treatments. TCM2COVID is freely available at http://zhangy-lab.cn/tcm2covid/.

## RESULTS

### Web interface

**Figure 2:**
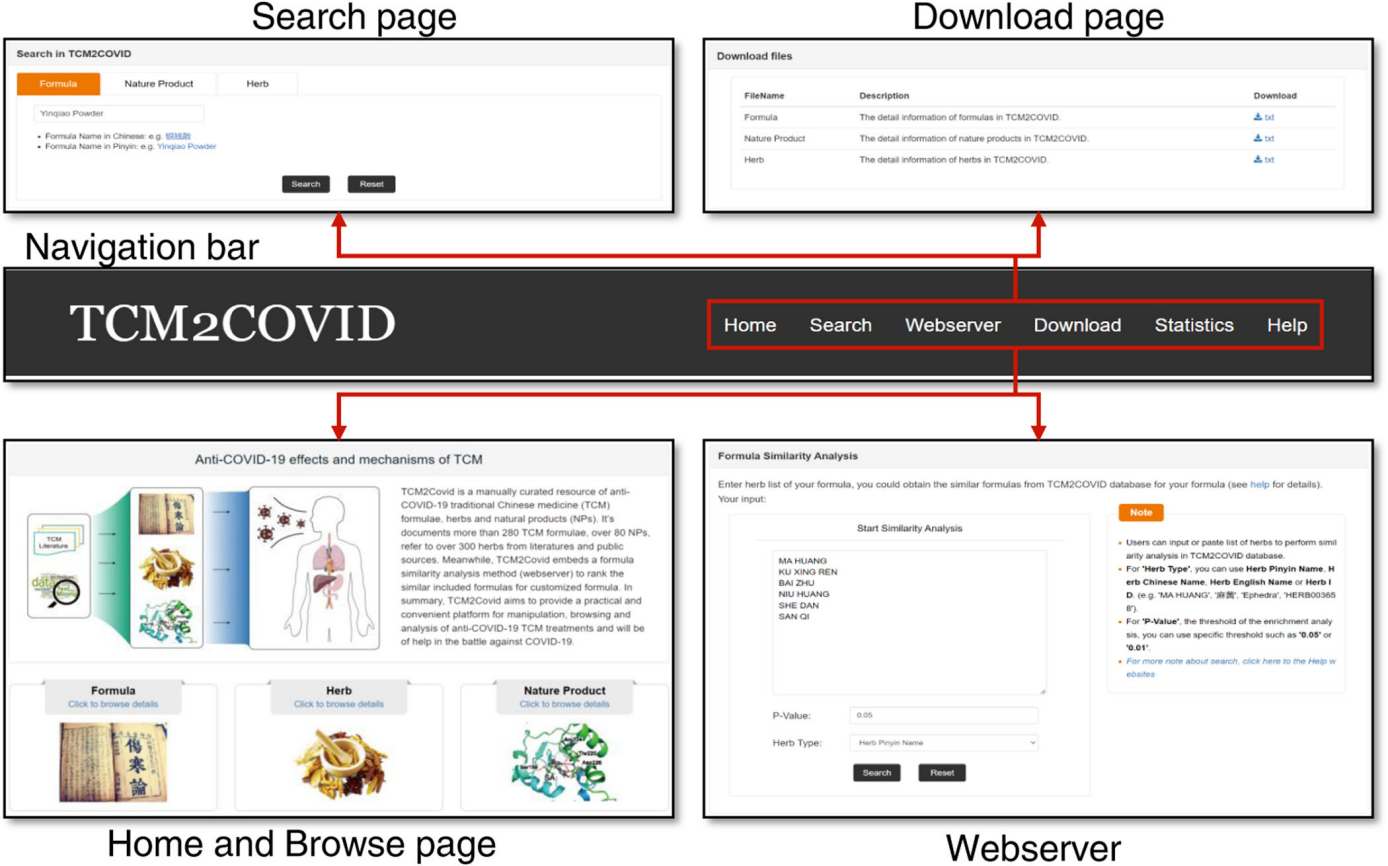
Web interface of TCM2COVID. Users can link to the Search, Download, Webserver, Statistics, and Help pages via the navigation bar.

TCM2COVID provides a convenient web interface that enables users to retrieve anti‐COVID TCM treatment data, and a web server has been developed for formula similarity analysis (Figure 2). Via the navigation bar, users can easily link to the Search, Download, Webserver, and Statistics pages. The Search page enables users to easily query TCM formulas, herbs, and NPs by inputting keywords (English, Chinese, Pinyin, or Latin name). On the Homepage, users also can browse the TCM formulas, herbs, and NPs data in the database by clicking on the corresponding hyperlink. The statistics for the TCM formulas, herbs, and NPs are presented on the Statistics page. All the data can be downloaded from the Download page. For user convenience, TCM2COVID features a Webserver page for formula similarity analysis. Users can upload herb lists of their own formulas to perform formula similarity analysis, and the results are presented as a bubble chart on the Result page. In addition, TCM2COVID provides detailed instructions and an example on the Help page.

### Data querying and results presentation

**Figure 3:**
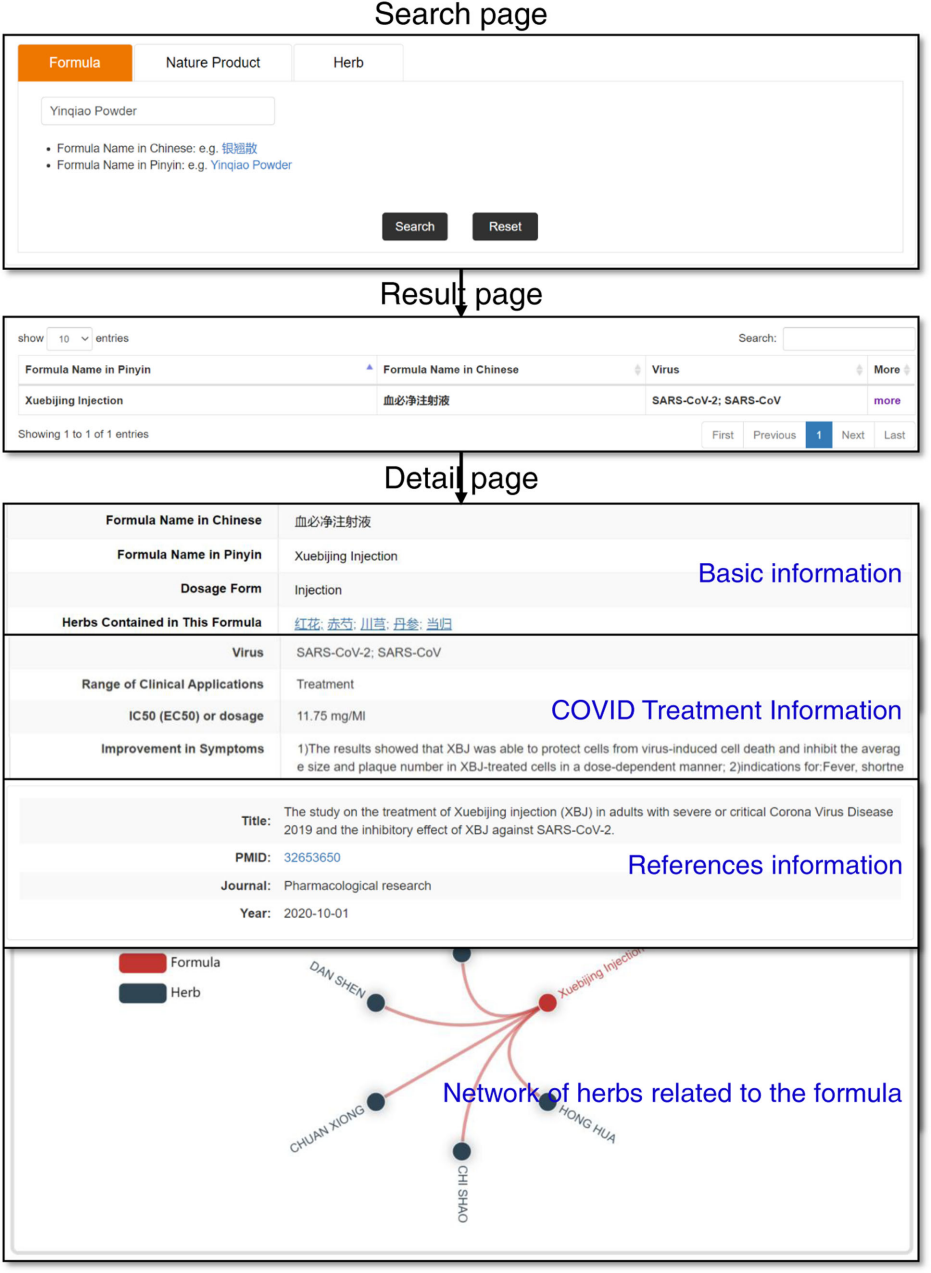
The Search page, Result page and Detail page (use Xuebijing Injection as an example)

To make it convenient for users to query and browse data, TCM2COVID provides three different search methods on the Search page, including “Formula Search” (searching a formula by inputting its Chinese name or Pinyin name), “Natural Product Search” (searching an NP by inputting its English name, Molecular formula, or PubChem CID) and “Herb Search” (searching a herb by inputting its English name, Chinese name, or Pinyin name) (Figure 3). A summary of the search results is presented in a table on the Result page. Detailed information on certain formulas, NPs, and herb entries can be viewed on the Detail page by clicking “more.” For formula entry, the Detail page presents basic information (name, dosage form, herbs contained in this formula, etc.), COVID Treatment Information (virus, IC50 (EC50) or dosage, improvement in symptoms, potential mechanism, etc.) and references information. For NP entry, the Detail page presents the basic information (NP name, 2D‐Structure, molecular formula, SMILES, original herbs, etc.), COVID Treatment Information (virus, effects, dosage, in silico method, etc.), and references information. For herb entry, the Detail page presents basic information (name, properties, meridians, indications, etc.) and a related ingredients list. Meanwhile, TCM2COVID provides an embedded network plot web tool on the Detail page to present the relationships between herbs and formulas or NPs. Users can highlight interactions of interest by moving the cursor over the diagram. Additionally, detailed instructions for querying, browsing, and analyzing anti‐COVID TCM treatments have been provided on the website of the database.

### Webserver for formula similarity analysis

**Figure 4:**
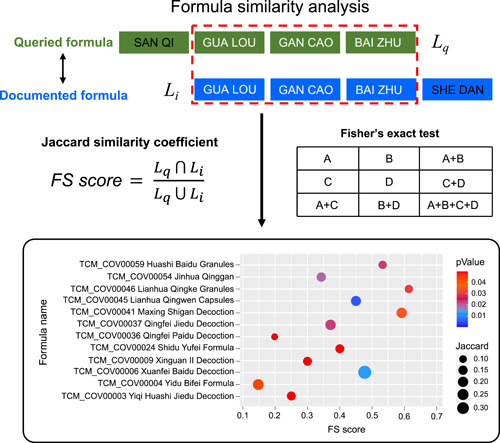
Schematic diagram of the webserver for formula similarity analysis

For user convenience, TCM2COVID launches a webserver for formula similarity analysis (Figure 4). First, users can fill out the herb list of the queried formula in the query window (inputting the herb's English name, Chinese name, Pinyin name, or ID). Users can also determine the threshold of the *p* value for Fisher's exact test. When the analysis is complete, the results are presented as a bubble plot on the Result page. The size of each bubble represents the *FS* score, and the color of the bubble represents the significance of the *FS* score. The results table containing *FS* scores and p values can be downloaded from the Result page.

## DISCUSSION

In China, TCM is playing an important role in the global fight against COVID‐19, and some studies begin to investigate the integrative mechanisms of TCMs by systematically analyzing the anti‐COVID‐19 TCM recipes [16, 30, 31]. Of which, Zhang et al. developed a comprehensive platform named soFDA, which integrated a network of disease–syndrome–TCM formula associations, and provided a series of elaborate web tools for TCM analysis. Users could query and analyze the potential COVID‐19 TCM formulas using the platform. However, a specialized database for storing and organizing anti‐COVID TCM treatments is still lacking. Herein, we developed TCM2COVID, a manually curated resource of anti‐COVID TCM formulas, NPs, and herbs. We believe that the TCM2COVID database will be a useful resource for the systematic studies and tools related to TCM and COVID‐19 therapy.

Although a series of TCM therapies have been approved to treat COVID‐19 [32, 33], the safety and effectiveness of TCM are still debated in the global medical field [34, 35, 36]. Due to a lack of high‐quality and rigorously peer‐reviewed clinical trials and poor understanding of the mechanism of action of TCM therapies, some studies suggest that the use of herbal drugs to treat COVID‐19 should be with caution [13, 35, 37]. Unfortunately, the argument has become political recently, making TCM an issue of political claims, which distorts what medicine should focus on and has hampered efforts to fight against COVID‐19 [38]. Despite this, there are many studies that have called for breaking down boundaries between Eastern and Western medicine and suggest that a proper combination of TCM and Western medicine could better control the disease progression and post‐disease syndromes [39, 40, 41, 42, 43, 44]. Herein, as part of the efforts for probing these issues, TCM2COVID, a comprehensive database, documents the detailed information on anti‐COVID TCM treatments and is undoubtedly a useful toolkit to help settle the controversy surrounding the effects and mechanisms of TCM therapies.

TCM2COVID still has some limitations. First, all formulas documented in the database are lacking information on herb proportion and importance (such as the principle of “monarch, ministerial, assistant and guide”), because most of original sources didn't provide these data. Meanwhile, some TCMs usually treat COVID‐19 in combination with western medicine, which is also an important part of the COVID‐19 treatments and should be collected in the database. Therefore, to remedy these data limitations, we will continue to collect and sort this information and update TCM2COVID in the future.

The current version of TCM2COVID documents TCM formulas and NPs from the literature and public sources. First, knowledge of the potential therapeutic mechanisms of the documented TCM therapies stored in TCM2COVID could provide suggestions for the pharmacological study and functional characterization of new anti‐COVID TCM treatments. Second, the continued accumulation of clinical trial data and outcomes of anti‐COVID TCM treatments will definitely provide the basis for clinical research and therapy for the disease. In addition, for user convenience, TCM2COVID launches a webserver for formula similarity analysis, which provides a useful platform for querying, analyzing, and visualizing documented formulas similar to those supplied by the user and thus definitely could be a useful tool for a basic and clinical study on new anti‐COVID TCM formulas.

## CONCLUSION

In summary, TCM2COVID documents over 280 TCM formulas (including over 300 herbs) with detailed clinical evidence and therapeutic mechanism information, and records over 80 NPs with detailed potential therapeutic mechanisms. It provides a user‐friendly and practical platform for documenting, querying, and browsing anti‐COVID TCM treatments and will help in the development and elucidation of the mechanisms of action of new anti‐COVID TCM therapies to support the fight against the COVID‐19 epidemic.

## METHODS

### Anti‐COVID TCM formulas

**Figure 5:**
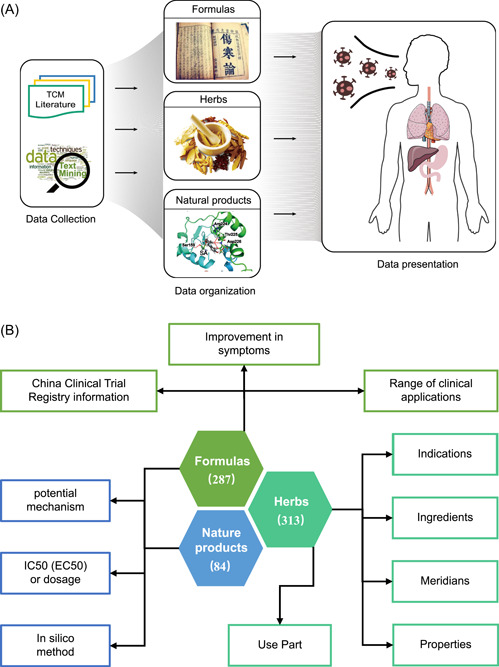
Collection, annotation, and organization of the TCM formulas, herbs, and NPs data. (A) Schematic of the database architecture. It contains data collection, data organization, and data presentation. (B) Statistics, annotation, and organization of the TCM formulas, herbs, and NPs data. NP, natural product; TCM, traditional Chinese medicine.

The TCM formulas in the TCM2COVID database were curated manually from peer‐reviewed studies and public sources, including national and local health commissions, hospitals, and TCM research institutes (Figure 5A). A total of 287 TCM formulas were collected, including 109 established TCM formulas, 143 formulas from the “Diagnosis and Treatment Protocol for COVID‐19” guidelines recommended by different health commissions, and 35 formulas prescribed by the “Master of TCM.” Detailed clinical evidence and therapeutic mechanism information, such as “potential mechanism,” “improvement in symptoms,” “IC50 (EC50) or dosage,” and “China Clinical Trial information (from Chinese Clinical Trial Registry (ChiCTR))” (Figure 5B), are also collected in the database. Moreover, 6 of the 287 TCM formulas have also been reported to be suitable for treating SARS.

### Anti‐COVID NPs

The NPs in the TCM2COVID database were curated manually from peer‐reviewed studies (Figure 5A). A total of 84 NPs were collected; of these, 48 NPs showed anti‐SARS‐CoV‐2 effects, 29 NPs showed anti‐SARS‐CoV effects, 2 NPs showed anti‐MERS‐CoV effects, and 4 NPs showed both anti‐SARS‐CoV and anti‐SARS‐CoV‐2 effects, and 1 NP showed both anti‐MERS‐CoV and anti‐SARS‐CoV‐2 effects. We also collected information on the “potential mechanism” and “IC50 (EC50) or dosage” for the anti‐COVID NPs from the literature (Figure 5B). Moreover, 14 NPs that have recently been explored by in silico methods (network pharmacology analysis, molecular docking, and virtual screening, etc.) are also documented in the TCM2COVID database.

### Anti‐COVID herbs

All the documented TCM formulas and NPs refer to 313 herbs. Information on “Properties,” “Meridians,” “Use Part,” “Indication,” and “Ingredients” was collected from the HERB database [45], SymMap database [46], TCMID 2.0 database [47] and ETCM database (Figure 5B) [48]. To unify the herbs and NPs from multiple resources into authoritative reference databases, the herbs in TCM2COVID were mapped to the HERB database (Herb id), and the NPs and the herb ingredients were mapped to the NCBI PubChem database (PubChem CID) [49].

### Pipeline for formula similarity analysis

For user convenience, TCM2COVID launches a webserver for formula similarity analysis (Figure 4). First, TCM2COVID quantifies the similarity between queried formula *q* and documented formula *i* using the formula similarity (*FS*) score, which is calculated by the Jaccard similarity coefficient [50, 51]:

(1) $$\mathrm{FS}\;\mathrm{score}=\frac{L_{q}\cap L_{i}}{L_{q}\cup L_{i}},$$

where *L*_*q*_ is the herb list of queried formula *q* and *L*_*i*_ is the herb list of documented formula *i*. Additionally, we estimated the significance of the similarity between the queried formula and the documented formula with Fisher's exact test.

### Architecture

TCM2COVID is implemented using the HTML and PHP languages with the MySQL server. The interface component consists of web pages designed and implemented in HTML/CSS. It has been tested in Google Chrome, Firefox, and Internet Explorer web browsers.

## AUTHOR CONTRIBUTIONS

*Conceptualization*: Yang Zhang, Kejun Deng, and Jian Huang. *Investigation*: Liping Ren, Yi Xu, Lin Ning, and Yuchen Li. *Coding*: Yi Xu and Yang Zhang. *Data collection*: Liping Ren, Xianrun Pan, Yang Zhang, Qi Zhao, Bo Pang, and Yuchen Li. *Writing*: Yang Zhang, Kejun Deng, and Jian Huang. *Funding acquisition*: Yang Zhang, Kejun Deng, and Jian Huang. All authors have read the final manuscript and approved it for publication.

## CONFLICT OF INTEREST

The authors declare no conflict of interest.

## Data Availability

All the data analyzed in the study are freely available at http://zhangy-lab.cn/tcm2covid/.
